# Manual therapists in Sweden during the COVID-19 pandemic -they remained in business, but how was their work environment and practice impacted?

**DOI:** 10.1371/journal.pone.0324245

**Published:** 2025-05-27

**Authors:** Iben Axen, Nathan Weiss, Eva Skillgate

**Affiliations:** 1 Institute of Environmental Medicine, Unit of Intervention and Implementation Research for Worker Health, Karolinska Institutet, Stockholm, Sweden; 2 The Norwegian Chiropractic Research Foundation “Et Liv I Bevegelse”, ELIB, Oslo, Norway; 3 Department of Health Promotion Science, Musculoskeletal and Sports Injury Epidemiology Center, Sophiahemmet University, Stockholm, Sweden; 4 Naprapathögskolan-Scandinavian College of Naprapathic Manual Medicine, Stockholm, Sweden; Caleb University, NIGERIA

## Abstract

**Background:**

The Swedish governmental strategy during the COVID-19-pandemic was to impose voluntary recommendations to limit viral spread, but to keep health care and important societal functions running. The objective of this study was to describe the work environment and practice of manual therapists, who were challenged by this strategy, in Sweden during a year of the pandemic.

**Methods:**

The cohort study Corona And Manual Professions (CAMP) was studying chiropractors and naprapaths, registered in the public register of licensed manual therapists in Sweden, during the pandemic. Mixed methods were used to answer the research aims. Surveys were distributed in November 2020, during the second wave, and in February, May and November of 2021. The quantitative data were presented descriptively, with the development over time illustrated in graphs. The qualitative data from the free-text answers were analyzed using content analysis.

**Results:**

In total, 816 manual therapists (47% of the invited sample) were included in the study, of which between 275 and 662 participants answered the free-text questions. At baseline, most (60–65%) rated their knowledge of viral infections and their spread, of vulnerable patient groups, and of protective gear as fairly good or good. Most (68–70%) were able to follow the official recommendations, but decreased numbers of patients and changes in clinic routines were reported. There was a positive trend in caring adequately for patients and having access to protective gear. Manual therapists reported that they were unable to care for vulnerable patient groups, had to adhere to routines perceived as onerous, and found care to be less personalized.

**Conclusion:**

At the time of the outbreak of the COVID-19 pandemic, manual therapists in Sweden encountered challenges regarding knowledge about pandemics and availability of protective equipment. Sweden’s official recommendations were possible to implement by the manual therapists, but had adverse impacts on clinic activities and patient care. Despite this, over 50% were able to deliver adequate care for their patients.

## Introduction

In March 2020, the COVID-19 -infection was declared a pandemic by the WHO [1]. Most countries around the world chose to impose restrictions, even lockdowns, on their citizens in an attempt to limit spread of the virus and subsequent burdens on the health-care systems, suffering and death.

Sweden chose a different strategy to get through the pandemic. In Sweden, limiting viral spread was based on voluntary compliance with official recommendations. These applied to all citizens and were not targeted to patient care. Congregations of people were limited (such as in theaters, concerts and at museums), a distance of two meters between people was recommended, and those who could were encouraged to work from home. A list of these recommendations are found in supplementary file 1. Schools and businesses were, however, open [2]. This strategy was chosen to uphold important societal functions such as health care and education and limit the potentially negative consequences of a lock-down, including loneliness and depression on the personal level, businesses going bankrupt on the corporate level, and the economy crumbling on a societal level.

Health care providers, such as dentists, manual therapists, psychologists and massage therapists, were allowed to remain in business and care for patients. Some restrictions were recommended for healthcare: careful cleaning and disinfecting, protective masks on both clinician and patient, and extra precautions regarding patients who were vulnerable to infections or who had any signs of present infection [3,4].

Manual therapists (in Sweden typically licensed chiropractors and naprapaths) differ from other health care providers as they use their hands when treating persons with musculoskeletal disorders, such as back and neck pain [5]. They are, therefore, physically close to their patients in the clinical encounter. The official recommendation of keeping a distance of two meters during the pandemic was not at all possible to uphold. Manual therapy normally doesn’t involve any bodily fluids and manual therapists are therefore not used to wearing any protective gear such as gloves and facemasks. Further, Swedish manual therapists practice mainly as owners of small private businesses. This translated to having to keep themselves updated regarding new measures to put in place as the pandemic evolved by relying on publicly available information to know what to do. Small clinics are more economically sensitive to a decreased flow of patients than bigger ones. Finally, it was estimated that approximately 50% of individuals infected with SARS-CoV-2 were asymptomatic or showed sub-clinical signs and symptoms [6,7], which may have caused involuntary exposure of infection for therapists as well as patients.

Research has yet to explore the impact of the Swedish COVID-19 strategy on the practice of manual therapists and their work environment while adhering to the recommendations. The objective of this study was to describe the work environment and practice of licensed manual therapists in Sweden during a year of the pandemic. This information may identify areas of improvement potentially relevant to the health of therapists as well as patients and to small businesses’ economy, and also to similar health care professions, such as physiotherapy. It was clearly a unique situation, and one that we may learn from in case of future pandemics.

Specifically, we aimed to study if the licensed manual therapists in Sweden during a year of the COVID-19 pandemic

1: felt they had adequate knowledge about the spread of infection, vulnerable patient groups and protection against a virus,
2: had access to adequate protection when delivering care,
3: were able to follow the official recommendations to reduce the spread of the virus when practicing,
4: changed the care of patients (as a result of official recommendations, worry or perceived need), and
5: could provide adequate care to their patients during this time.

Further, we explored differences between therapists with short- and long work-experience, and whether these items changed during a year of the pandemic.

## Methods

This cohort study is based on the Corona And Manual Professions (CAMP)-study, which was set up to study the impact of the pandemic on Swedish chiropractors and naprapaths (Clinical Trials register identifier: NCT04834583, registered 08/04/2021.). The study procedures are described in detail elsewhere [8]. Mixed methods were used in this study where both quantitative and qualitative data were used to answer the research aims. The questions relating to work environment and clinical practice were designed by the research group and presented to a focus group of manual therapists before distribution, and face validity was deemed to be good.

### Selection and description of participants

We invited all individuals in the public register of licensed manual therapists in Sweden and included those who were clinically active (defined as working with health promotion, prevention and or treatment) at the time of the distribution of the first survey, in November 2020. Thus, the size of the source population was known in advance. The potential participants were sent an email with information about the study, and a link to the informed consent and the digital baseline questionnaire.

The study collected data on manual therapists’ health, work environment and economy four times through the second and third wave of the pandemic; in November 2020, February 2021, May 2021 and November 2021, with a response rate of 80% at each follow-up. This study reports on questions related to the work environment. Specifically, we asked about prior knowledge about infections, vulnerable patient groups and the impact of the official recommendations (if the manual therapists were able to comply, and the impact on patient care). Further, we investigated the knowledge and availability of protective equipment.

All data were self-reported through digital questionnaires, including free-text options where the respondents could address a subject in their own words.

### Ethics

The study received ethical permission from the Swedish Ethical Review Authority Dnr: 2020–03836. All participants provided an informed consent to participate by ticking a box in the questionnaire stating “I have understood what this study entails and consent to participate”.

### Statistics

The quantitative data were summarized with means and standard deviations (SD), and the development over time was illustrated in graphs, using Excel.

The qualitative data from the free-text answers were analyzed using content analysis [9]. The answers were exported to Excel and read though twice by two of the co-authors to get an overview of the content. Then the text was manually divided and condensed into subcategories. Based on these, categories were identified and labeled after a discussion with the research group.

## Results

The public list of licensed manual therapists in Sweden consisted of 1718 individuals, after excluding those who were retired, not working in Sweden, or who were not clinically active in November 2020. Of those, 816 (47% of the invited sample) filled in the baseline questionnaire and were included in the study. The sample is described in Table 1. Gender distribution was even, the mean age was 44 years, two-thirds were naprapaths, and about half had been practicing for more than 15 years.

**Table 1:** Description of the cohort (n = 816) of clinically active manual therapists.

Variablel	Category	N = 816
Gender: % (n)	Male: Female: Other:	54 (440) 46 (375) (1)
Age, years, mean (SD) Age, median (min, max)		44.0 (11.2) 43.0 (23.0, 74.0)
Profession; % (n) Chiropractor Naprapath		32 (262) 68 (554)
Years in practice: % (n)	< 10 years: 11-19 yrs: 20-29 yrs: >29 yrs: Missing:	31 (240) 28 (214) 24 (186) 18 (136) (40)
	<=15 yrs: >15 yrs: Missing:	50 (406) 50 (407) (3)
Number of hours clinically active/week % (n)	< 20 hrs: 20-40 hrs: >40 hrs: Missing:	12 (95) 79 (625) 9 (75) (21)
Other employment: % (n)Extent in % of full time: mean (SD) Median (min, max)	Yes: No: Missing	22 (175) 78 (638) (3)
	46 (31) 40 (1, 100)
Number of colleagues working together; % (n)	Working alone: 2: 3-4: 5 or more: Other: Missing:	22 (175) 14 (116) 26 (207) 36 (290) 3 (22) (6)
Business owner: % (n)	Solo owner: Part owner: Renting: Employed: NA: Missing:	40 (318) 22 (179) 12 (100) 25 (206) 1 (7) (6)
Type of payment of patients -several options possible	Private: Sports teams: Businesses: County council: Insurance: Other:	771 373 499 113 583 22

The majority worked between 20 and 40 hours per week, and an even distribution between working alone, together with one, two or more colleagues was observed. Of the sample, about half were owners of their clinic, either alone or in joint ownership. The majority of patients were paying out of pocket, but many were also reimbursed by insurance or through business deals.

In Table 2, the manual therapists’ perceptions about their status prior to the pandemic is reported. Most (65%) rated their knowledge of viral infections and their spread as fairly good or good, with similar values for those with short (< 15 years) and long (>15 years) clinical experience (estimates not shown). The same was found regarding knowledge of vulnerable patient groups, of protection and protective gear; the majority (over 60%) rated their knowledge as fairly good and good, again with similar values for those with short and long working experience (estimates not shown). In an overall rating, equal numbers rated their prior knowledge about pandemics as adequate as not adequate.

**Table 2:** Results related to aim 1: Knowledge about the spread of infection, vulnerable patient groups and protection against a virus, and protective gear, among manual therapists in Sweden, prior to the pandemic.

Questions	Answer categories	% (n)
General knowledge regarding viral spread and protection: Missing (12)	Very good: Good: Fair: Poor: Very poor: Not clinically active^*^	14 (113) 43 (345) 32 (255) 8 (63) 3 (21) (7)
General knowledge of vulnerable patient groups: Missing (12)	Very good: Good: Fair: Poor: Very poor: Not clinically active^*^	14 (115) 41 (327) 33 (269) 9 (73) 2 (13) (7)
Overall adequate knowledge of epidemics/pandemics: Missing (12)	Yes: No: Not clinically active:	53 (428) 45 (361) 2 (15)
Knowledge of how to protect myself and my clients: Missing (15)	Very good: Good: Fair: Poor: Very poor: Not clinically active^*^:	9 (70) 40 (317) 35 (278) 12 (99) 2 (20) 2 (17)
Knowledge of protective gear: Missing (12)	Very good: Good: Fair: Poor: Very poor: Not clinically active^*^:	6 (51) 31 (249) 42 (333) 18 (140) 3 (21) 1 (10)

* The sample included manual therapists who were clinically active at baseline. However, some had not been working during the previous 3 months and could not reply to questions pertaining to this time.

**Fig 1:**
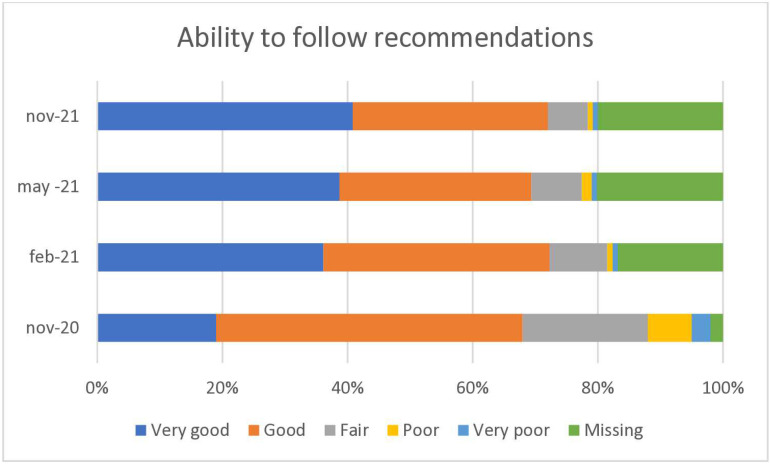
Manual therapists in Sweden are rating their ability to follow the official recommendations in November 2020, February 2021, May 2021 and in November 2021.

**Fig 2:**
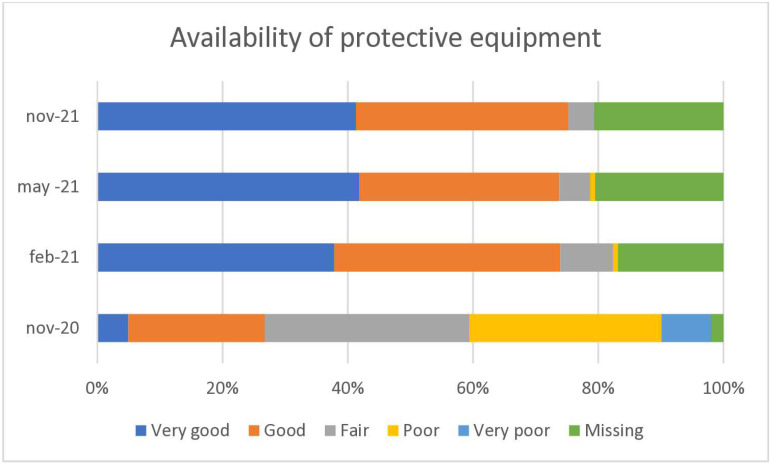
Manual therapists in Sweden are rating the availability of protective equipment in November 2020, February 2021, May 2021 and in November 2021.

**Fig 3:**
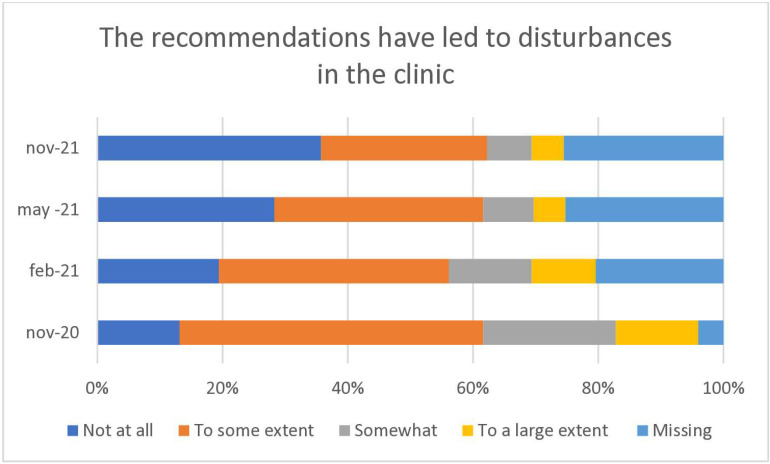
Manual therapists in Sweden are rating if the official recommendations led to disturbances in the clinic in November 2020, February 2021, May 2021 and in November 2021.

**Fig 4:**
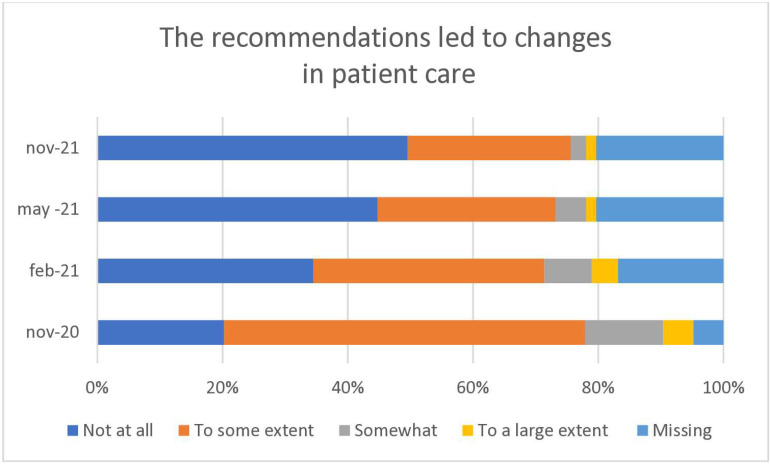
Manual therapists in Sweden are rating if the official recommendations led to changes in patient care in November 2020, February 2021, May 2021 and in November 2021.

**Fig 5:**
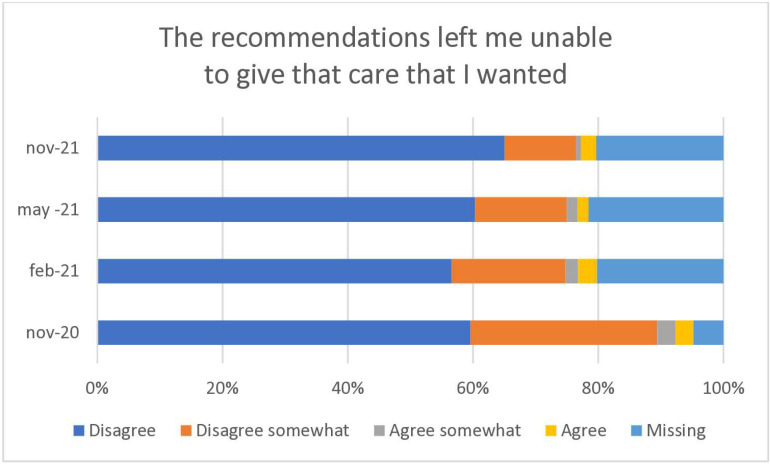
Manual therapists in Sweden are rating if the official recommendations left them unable to deliver patient care as they wanted in November 2020, February 2021, May 2021 and in November 2021.

In Figs 1–5, related to aims 2, 3 and 4, manual therapists’ access to adequate protection, perceptions of the official recommendations and their impact was described, at baseline (November 2020), after 3 months (February 2021), 6 months (in May 2021) and after one year (November 2021).

At the start of the study, only a fifth of manual therapists were able to follow the official recommendations very well, but this doubled over time (Fig 1). At baseline, a fifth stated they were able to follow the official recommendations poorly or very poorly, and this decreased to 1% at the last follow-up.

At baseline, 35% of the respondents stated that the availability of protective equipment was poor or very poor, and this dropped to 1% after one year (Fig 2).

The picture was similar concerning disturbances in clinic due to the official recommendations, a third stated that they disturbed clinic quite a lot or to a large extent at baseline. After one year, this proportion had dropped to 10% (Fig 3).

The official recommendations led to changes in patient care to some extent for the majority (58%) of clinics at baseline, a figure that was halved during the year of the study (Fig 4).

However, throughout the year of the study, half of the respondents claimed that they, despite the official recommendations, could deliver adequate care, and only 2% stated they could not (Fig 5).

To further explore the questionnaire results, we provided the respondents with an opportunity to explain the reasoning behind their responses, with three free-text questions in the baseline questionnaire. Even though the space for writing was unlimited in the digital format, most respondents offered short statements or keywords only. The resulting categories were summarized in Table 3, and examples of statements were given.

**Table 3:** Free-text questions regarding the impact of the official recommendations, the resulting subcategories and categories.

Questions	Subcategory	Category
In what way have the official recommendations led to disturbances in your clinic? (n = 660)	• More cancellations. • Fewer new patients. • People working from home. • Avoiding public transport. • Uncertainty about safety with manual therapy. • Associated businesses closed. • Short-notice cancellations due to symptoms. • Fewer patients from vulnerable groups.	Decrease in patient numbers
	• Increased cleaning • Use of protective gear. • Adapt waiting room for social distancing. • Start tele/video consultations.	Change in clinic routines
In what way have the official recommendations changed patient care? (n = 662)	• Elderly not treated. • Accommodate clinic routines for vulnerable patients groups. • start tele/video consultations.	Inability to care for vulnerable patient groups
	• Disinfection. • Social distancing.	Perceived onerous routines
	• Stopped greeting patients with handshake. • Protective masks hindering communication. • Changed manual techniques. • Start tele/video consultations.	Less personalized care
How has the official recommendations hindered you from giving the care that you wanted? (n = 275)	• Poor “doctor-patient” relationship. • Techniques not always optimal for the patient’s complaint. • Patients unable to follow treatment plans.	Sub-optimal care conditions

The first free-text question was “*In what way have the official recommendations led to disturbances in your clinic?*”. Our interpretation of the 660 answers was that the most common disturbance was a decrease in patient numbers, and thus poorer economy, Thus the first code was “*Decrease in patient numbers*”. This decrease was perceived as being due to patients’ fear of getting infected, and that they were abiding by the recommendations of avoiding public transport, working from home, and only socializing with family members. Example of statements were:

“Fewer patients, due to the authorities’ recommendations to stay at home as much as possible.”

“Economic loss due to fewer bookings because of a fear of getting infected.”

Also commonly reported was a decrease in the number of patients with a high risk of complications from getting infected, like the elderly. Another factor mentioned as leading to a decrease in patient numbers was the recommendation to stay at home when there was any risk of being infected. The clinics had to accept cancellations on short notice, which clearly affected their economy. Manual therapists working at corporate businesses or sports clubs experienced a total stop in their patient flow as these types of businesses closed temporarily. Some manual therapists reported that they started giving tele/video-consultations to accommodate patients that were unable to come to the clinic for care.

The second code was “*Change in clinic routines*“and described the extra precautions relating to cleaning, disinfection, wearing protective gear and accommodating vulnerable patient groups.

The second free-text question, answered by 662 individuals, was “*In what way have the official recommendations changed patient care?*”. The major point here was the inability to give care to the elderly and other vulnerable patient groups (code: “*Inability to care for vulnerable patient groups”*). Some respondents stopped treating these patients altogether, and some accommodated them with special slots in the clinic (when no other patients were around) or engaged in tele/video-consultations with these and other patients. One participant wrote:

“Vulnerable patient groups are seen at designated times to minimize the contact with others and allow extra thorough cleaning before and between visits.”

The burden of disinfection and protection (code: “*Perceived* o*nerous routines* “) was mentioned in the context of hindering the “doctor-patient” relationship. Handshakes were abandoned, and facemasks were described as hindering communication, both verbal and non-verbal (code “*Less personalized care*”). Many manual therapists explained how they selected techniques that were perceived as involving less risk of viral spread: i.e., mainly using techniques with the patient lying face down. An example was:

“Do not shake hands, keep a distance while taking the anamnesis, facemask and sometimes face-shield. Feels less personal.”

Lastly, we asked “*How has the official recommendations hindered you from giving the care that you wanted?*” answered by 275 individuals. It was quite clear that the measures described above were perceived as rendering sub-optimal patient care (code “*Sub-optimal care conditions*”) and treatment results. Again, the poorer “doctor-patient” relationship as a result of not shaking hands and wearing a facemask, was seen as a barrier to a good treatment response. Selecting manual technique on the basis of patient positioning to minimize viral spread and not patient condition, was perceived as a clear reason for not getting optimal treatment results. Finally, as patients wanted to minimize clinic time, they often did not follow their treatment plans, and cancelled follow-up. One participant replied:

“I have chosen to not use certain techniques that may be more effective, to avoid close contact. Have to carefully communicate what I am doing to avoid talking “over” the patient.”

## Discussion

Clinically active licensed manual therapist in Sweden were followed during one year of the COVID-19 pandemic in this cohort study. Their knowledge of viral infections and their spread, how to protect themselves and vulnerable patient groups in case of infectious diseases, and knowledge about protection and protective gear was mostly reported as “fairly good” or good”. Knowledge about pandemics before COVID-19 was rated as “adequate” equally often as “not adequate”. Further, the availability of protective equipment was first limited but improved as the pandemic progressed. These results did not differ between therapists with short and long work-experience.

We aimed to understand the consequences of the official recommendations on the work environment, as perceived by manual therapists. A majority reported at study start that they were able to follow the official recommendations, and this answer became more common over time. Disturbances in clinical activities due to the official recommendations were more commonly reported than changes in patients care for the same reason but became less common over time. However, throughout the year of the study, half of the respondents claimed that they, despite the official recommendations, could care adequately for their patients.

To deepen the understanding of the impact of the official recommendations, three free-text questions were asked. Concerning how the official recommendations led to *disturbances* in the clinic, several factors emerged, coded as “decreased number of patients”, and “changes in clinic routines”. Analyzing the answers of how the official recommendations *changed patients care*, the categories “inability to care for vulnerable patient groups”, “perceived onerous routines”, and “less personalized care” emerged. Sub-optimal conditions like poor “doctor-patient” relationship and that the treatment techniques were sub-optimal were brought up when the question about how the official recommendations *hindered* the therapists from giving the care they wanted.

The knowledge about viral infections and their spread, and protection against infection was perceived to be good by half of this population, but it was common not to have knowledge about pandemics. This was not surprising as this group of caregivers are used to handling patients’ common viral and bacterial infections such as hepatitis, influenza, and strep throat. However, a pandemic has not been a challenge in recent times. In the upcoming years, the recent COVID pandemic will be remembered by all those who lived through it, including healthcare personell, who then will have such knowledge.

It was also expected that the availability of protective equipment was limited early in the pandemic, but gratifying to note that it improved as the pandemic progressed. In a Swedish report of health care workers during the COVID-19 pandemic, this finding was echoed [2]. It is important for the professional associations and clinic managers to consider this knowledge and find ways to improve the preparedness for coming pandemics.

In Sweden, the work environment of personnel working in hospitals and care-homes during the pandemic has been evaluated: One of the areas found to have been compromised was the lack of protective gear [2]. Thus, manual therapists seem to have struggled with the same problem.

The manual therapists generally reported that they were able to follow the official recommendations concerning keeping a distance and strict hygiene, and the adherence to recommendations improved over time. This was despite the challenging situation for manual therapists using their hands and being physically close to their patients when treating musculoskeletal disorders. In a study from Sweden examining maternal healthcare, the adherence to the official recommendations was described in terms of increased job demands and high workload [10]. In this study, collegial support was mentioned as of major importance during the pandemic. This is something that is unavailable for many manual therapists, as they are working alone.

Our results are not easily compared to other studies of healthcare personnel during the pandemic from other areas of the world, where lockdowns were typically enforced, but they may be contrasted. In a small international survey, physiotherapists reported a shortage of protective gear and adapting their manual treatment, similar to our findings [11]. In a US study examing surgeons’ training during the pandemic, optimization of virtual platforms, prioritizing mental well-being, and the necessity of developing strategies to mitigate the impact of future disruptions was stressed [12]. In a Spanish study, physiotherapists found that their patients’ function declined due to the lack of care and to changes in treatment protocols as a result of lockdown [13], and also here, the need for improved technological literacy was mentioned to mitigate such effects in a future pandemic.

In Sweden, there was a concern by the professional associations for the survival of manual therapists’ businesses due to the official recommendations. The result of this study showed that this concern was justified. The majority of the participants reported that the recommendations led to decreased patient numbers, as well as changes in the care provided, such as the inability to care for vulnerable patient groups and less personalized care. Sub-optimal conditions highlighted were poor “doctor-patient” relationship and not being able to use the most optimal treatment techniques. However, with time, it seemed that these therapists found ways to run their businesses despite the recommendations, suggesting that the therapists found a balance between societal and clinical needs. When studying the economy of the manual therapists, we have previously reported how business owners enforced strategies to manage decreased income in order for their business to survive [14].

Nevertheless, only half of the caregivers over a year of the pandemic reported that they could give the care they wanted to their patients. This indicated that the pandemic had a large impact on these caregivers and by extension probably also on their patients. This is similar to a Swedish report where health care personnel described worrying about the quality of their work under these circumstances [2].

Thus, it seems that Sweden’s strategy to issue official recommendations to limit the spread of the virus but not proceeding to lockdown, led to a difficult but manageable situation for manual therapists. The change in the context of care delivery, changing the work environment and routines to adhere to the recommendations, affected patient care but became easier over time. To support manual therapists working alone in small private clinics during crises as a pandemic, strategies to enable collegial support to manage challenges related to the safety of patients as well as care givers, may be important health policy actions.

It is a methodological strength that the study was based on a large sample (n = 816) and that the sample constituted 47% of the total population of clinically active licensed chiropractors and naprapaths in Sweden at the time. This strengthens the external validity of our findings. It is not likely that the results are generalizable to manual therapist outside Sweden since the official recommendations in Sweden during the COVID-19 differed significantly from other countries. Further, the results may not easily be compared to the situation of caregivers working in publicly funded settings in Sweden. Some of the results – especially regarding knowledge about pandemics before the outbreak of the COVID-19, and the availability of protective gear might be generalized to other small private health care providers in Sweden, that similarly to the caregivers in this study had limited access to updated information and equipment.

Even though a large proportion of the clinically active licenced manual therapists was included in the study, it cannot be ruled out that the answers and statements reported are over- or under-estimated, as not all manual therapists participated in the cohort study. If the non-responders differed from the responders, for instance in being able to run their business during the pandemic, we might have drawn the wrong conclusions.

Some questions were asked repeatedly over a year to map changes during the pandemic. However, 20% of the respondents dropped out during the year of data collection, even though a follow-up rate of 80% is considered to be relatively high. If the responders differ from those not responding, we may have misjudged changes over time.

Most of the questions in the questionnaire were developed by the research group for the purpose of this study, and the psychometric properties are unknown. The questions were deemed to have good face validity but might have been misinterpreted. Still, the free-text responses and the mixed model method improved the possibility to fulfill the aim of the study.

We consider the qualitative part of the study to be trustworthy [15] as the experiences described in our study resonates with members of the research group, who are manual therapists living through the pandemic in Sweden. To ensure credibility, the free-text questions were reviewed in a focus group with a convenience sample of licensed naprapaths and naprapath students. The survey was sent in advance to focus group members who then were invited to participate in an audiotaped meeting; whereupon they were asked about the survey’s comprehensibility and potential improvements.The focus group discussions led to minor wording revisions to improve clarity and interpretability. Participants were given opportunities to refuse to answer any question, which ensured that only participants who were genuinely willing to offer information participated. Further, the researchers continuously evaluated the data collection through a reflective approach, the participants had the opportunity to comment on structure of the survey at each time-point. Considering the sample comprises a large proportion of the clinically active licensed manual therapists at the time of data collection, the results may be transferable to other manual therapists during this time. However, considering Sweden’s unique strategy of handling the COVID-19 pandemic, the ability to transfer these results to manual therapists in other countries or contexts might be limited. The use of mixed-method comprising both quantitative and qualitative data might improve the dependability of the study. Lastly, the use of a manifest content analysis with categories formed with very close ties to the raw text as possible, with minimal interpretation from the researchers in the analysis phase ensured confirmability of the results [16].

## Conclusion

At the time of the outbreak of the COVID-19 pandemic, manual therapists in Sweden encountered challenges regarding knowledge about pandemics and availability of protective equipment.

Sweden’s official recommendations were possible to implement, also for care that normally requires close patient contact as most therapists reported being able to deliver adequate care for their patients within a year. These findings may be of potential importance to optimize regulations to balance safety and the need for care delivery to reduce the impact of potential future pandemics on society.

## Supporting information

S1 File The voluntary recommendations that were enforced by the Swedish Authorities during the first year of the COVID-19 pandemic. (DOCX)
