# Supplementary material for: Manual therapists in Sweden during the COVID-19 pandemic -they remained in business, but how was their work environment and practice impacted?
Source: PLoS One. 2025 May 27;20(5):e0324245. doi: 10.1371/journal.pone.0324245 (PMC12112402; doi:10.1371/journal.pone.0324245)
Supplement: S1 File — (DOCX) [file pone.0324245.s001.docx]

| **Date**  Supplementary file 1: The voluntary recommendations that were enforced by the Swedish Authorities during the first year of the COVID-19 pandemic.  Source: [https://www.sciencedirect.com/science/article/pii/S2352340921009732#section-cited-by](https://eur01.safelinks.protection.outlook.com/?url=https%3A%2F%2Fwww.sciencedirect.com%2Fscience%2Farticle%2Fpii%2FS2352340921009732%23section-cited-by&data=05%7C02%7Ciben.axen%40ki.se%7Cefa1cf1b3cd94c469db308dd4cef42fd%7Cbff7eef1cf4b4f32be3da1dda043c05d%7C0%7C0%7C638751312700300830%7CUnknown%7CTWFpbGZsb3d8eyJFbXB0eU1hcGkiOnRydWUsIlYiOiIwLjAuMDAwMCIsIlAiOiJXaW4zMiIsIkFOIjoiTWFpbCIsIldUIjoyfQ%3D%3D%7C0%7C%7C%7C&sdata=D0aMnaY%2Byu2rxA2mjeHQMv%2FopCvrYKbmiXb8rFzoy4o%3D&reserved=0) | **Principal Actors** | **Event/recommendation** |
| --- | --- | --- |
| 31 Jan 2020 | Patient | First confirmed case of COVID-19 in Sweden. |
| 6 Mar 2020 | Swedish Government | Advice against all unnecessary travel to parts of northern Italy and South Korea. |
| 10 Mar 2020 | Swedish National Board of Health and Welfare | Signs of community spread of COVID-19 in Sweden. |
| 10 Mar 2020 | Swedish Public Health Agency | People experiencing COVID-19 related symptoms should not work with elderly or people in people in risk groups. |
| 11 Mar 2020 | Swedish Public Health Agency | Everyone with even mild cold or flu symptoms should stay at home to limit the spread of the virus. |
| 11 Mar 2020 | Swedish Government | Prohibitions against public gatherings and public events of more than 500 participants. |
| 12 Mar 2020 | Swedish Public Health Agency | Schools are to remain open. |
| 13 Mar 2020 | Swedish Public Health Agency | Anyone experiencing flu-like symptoms should undertake voluntary quarantine while available testing capacity. |
| 13 Mar 2020 | Swedish National Board of Health and Welfare | Information on basic hygiene and other measures that can limit the spread of COVID-19 in care and healthcare. |
| 14 Mar 2020 | Swedish Government | Anyone planning to travel abroad should carefully consider the risks. |
| 15 Mar 2020 | Swedish Public Health Agency | Avoid unnecessary visits to hospitals or homes for the elderly and maintain good hand hygiene. |
| 16 Mar 2020 | Swedish Public Health Agency | Employers should allow personnel for which it is possible to work from home. People aged 70 and above are to avoid close social contacts. |
| 17 Mar 2020 | Swedish Public Health Agency | Education for children over 16 and adults (e.g., high schools, universities, university colleges, and adult vocational education) move to distance and online learning. |
| 24 Mar 2020 | Swedish Government | All members of the public are to practice social distancing and to telephone friends and relatives rather than meet with them in person, especially important for people in risk categories such as elderly people. |
| 25 Mar 2020 | Swedish Public Health Agency | Sport practices, matches, and local cups do not need to be cancelled due to the COVID-19 pandemic. Gyms, swimming pools and sports halls may also stay open. |
| 27 Mar 2020 | Swedish Public Health Agency | Ban on public gatherings of more than 50 people. |
| 30 Mar 2020 | Swedish Government | A national ban on visiting all of the country's elderly care home. |
| 1 Apr 2020 | Swedish Public Health Agency | Larger contexts should be avoided where several people meet, such as parties, weddings and other events. It is also important that people keep a distance from one another at, for example, sports venues, gyms, shopping malls, on public transport and other locations. People who are over 70 or who belong to other risk categories should limit their social contact with other people, avoid public transport and avoid shopping in pharmacies, supermarkets or crowded places. |
| 3 Apr 2020 | Swedish Public Health Agency | Recommendation against non-essential travel to all countries outside Sweden. |
| 4 Jun 2020 | Swedish Government | Symptom-free individuals are allowed to travel domestically. |
| 30 Jul 2020 | Swedish Public Health Agency | People who are able to work from home continue to do so in the fall. Teleworking contributes to reducing crowding, including on public transport. |
| 22 Sep 2020 | Swedish Public Health Agency | Sports participants, organizations, and organizers are to ensure that recommendations on distancing, hand hygiene, and voluntary quarantine if experiencing COVID-19 related symptoms are followed at sport events. |
| 29 Oct 2020 | Swedish Public Health Agency | “We are asking both employers and employees to do their best to make it possible to work from home as much as possible.” |
| 3 Nov 2020 | Swedish Public Health Agency | Dining parties will be reduced to a maximum of eight people, audiences at concerts and similar events must be seated, and businesses are recommended to limit the number of patrons allowed on premises. |
| 9 Nov 2020 | Swedish Government | Advice against all non-essential travel to countries outside the EU/EEA/Schengen area and the UK. |
| 12 Nov 2020 | Swedish Public Health Agency | Schools are not a risk environment in terms of COVID-19 infections. |
| 26 Nov 2020 | Swedish Government | Avoid shopping during peak hours. |
| 1 Dec 2020 | Swedish Public Health Agency | Symptom-free children should stay home from school if someone in their household has tested positive for COVID-19. |
| 3 Dec 2020 | Swedish Public Health Agency | Upper secondary schools are advised to partially close and switch to distance learning. |
| 8 Dec 2020 | Swedish Public Health Agency | Keep private gatherings during the Christmas season small and within the eight people limit, only meet for a short time, and hold gatherings outdoors if possible and to maintain physical distancing. |
| 17 Dec 2020 | Swedish Public Health Agency | Pregnant people should take additional precautions in relation to the COVID-19 pandemic. |
| 18 Dec 2020 | Swedish Transport Agency | All motorists should take precautions and maintain a safe distance so that to minimize the risk of accidents on the busy Christmas roads. |
| 22 Dec 2020 | Swedish Public Health Agency | Businesses should determine a maximum number of patrons to be allowed on premises at any given time, in shops, shopping centers and fitness centers, according to size, furnishing and ventilation capacity. |
| 30 Dec 2020 | Swedish Public Health Agency | Face masks to be worn on public transport lacking reserved seating and during rush hour. |
| 8 Jan 2021 | Swedish Public Health Agency | Municipalities should consider opening up outdoor venues for youth sports in order to limit the negative effects to children's health as public venues such as sporting facilities have been closed down. |
| 2 Feb 2021 | Swedish Public Health Agency | Astra Zeneca vaccine to be given primarily to people aged 18 to 64 while people aged 65 and older receive one of the previously approved vaccines from Pfizer/BioNTech or Moderna. |
| 25 Feb 2021 | Swedish Public Health Agency | Vaccinated seniors may now return to a slightly more normal life with regard to contact with other people. |
| 15 Apr 2021 | Swedish Public Health Agency | Self-reported adherence to restrictions and recommendations is falling among Swedish residents, who are strongly encouraged reverse this trend and improve adherence. |
| 21 Apr 2021 | Swedish Public Health Agency | Only healthcare and care staff who in their work interact with and spend time in proximity with patients and who thereby may contract and spread COVID-19 are to be prioritized in the vaccination program. |
| 28 Apr 2021 | Swedish Public Health Agency | Children born 2002 or later may participate in occasional sporting competitions or games if organized locally or with restrictive travelling and if limited to one game or competition per week. |
| 24 May 2021 | Swedish Government | Advice against non-essential travel. |
| 27 May 2021 | Swedish Public Health Agency | Adult education can move back to campuses after three semesters of distanced and online learning. |
